# The role of intersectionality in shaping participant engagement with health research through digital methods: findings from a qualitative study

**DOI:** 10.1186/s13063-025-08929-0

**Published:** 2025-06-21

**Authors:** Cherish Boxall, Felicity L. Bishop, Nisreen A. Alwan, Shaun Treweek, Gareth Griffiths, Nnenna Ekeke, John McGavin, Jane Thorp, Katherine Bradbury

**Affiliations:** 1 School of Psychology, University of Southampton, Southampton, UK; 2 Southampton Clinical Trials Unit, University of Southampton, Southampton, UK; 3 School of Primary Care, University of Southampton, Southampton, UK; 4 University Hospital Southampton NHS Foundation Trust, Southampton, UK; 5 NIHE Applied Research Collaboration Wessex, Southampton, UK; 6 Aberdeen Centre for Evaluation, University of Aberdeen, Aberdeen, UK; 7 National Health Service East of England, Cambridge, UK; 8 Public Partners, Southampton, UK

**Keywords:** Digital, Recruitment, Retention, Intersectionality, Equity, Qualitative

## Abstract

**Background:**

Digital research methods were rapidly adopted into clinical trials and health research during the COVID pandemic in 2020. Current UK policy aims to make digital research methods a norm, but their influence on recruitment, retention, and representation in health research remains largely unknown. Whilst efforts have been made to improve engagement with digital health interventions, less attention has been given to digital research methods—such as informed consent, data collection, and research communications—despite their potential to influence study participation and participant experience.

**Objective:**

This qualitative study aims to understand the factors influencing the initial uptake and ongoing engagement with digital research methods across diverse populations, capturing experiences and perspectives to inform diverse and efficient health research conduct.

**Methods:**

Semi-structured interviews were conducted with 50 people who had participated in health research in the past 12 months. Reflective thematic analysis was used to understand factors influencing study engagement from participant perspectives, acknowledging the role of the researcher in data interpretation.

**Results:**

Three interconnected themes were identified: (1) Digital Positionality: The Interplay of Social Position, Personal Experience, and Identity; (2) Power Redistribution in Research Relationships: Navigating Vulnerability and Agency; (3) Trust Assemblages: How Intersecting Identities Shape Multi-modal Verification Practices in Research Engagement. These themes illustrate how intersecting identity factors and social contexts shape engagement with digital methods in health research. The first theme revealed how factors such as age, social role, migration, and socioeconomic status create pathways towards or away from engagement with digital methods. The second theme highlights how different digital methods can shift power dynamics in participant-research relationships or expose social vulnerabilities. The third theme uncovered the complex ways participants established trust in research, relying on multi-channel trust makers.

**Conclusions:**

The study reveals intersecting factors shaping participant engagement with digital methods, offering insights to enhance research conduct and increase diversity in health research participation. Future studies should integrate theoretical frameworks to examine these influencers and develop effective approaches for optimising diverse engagement with digital methods.

**Supplementary Information:**

The online version contains supplementary material available at 10.1186/s13063-025-08929-0.

## Introduction

From 2020 to 2022, the use of digital methods (Table 1) to conduct health research increased at a rapid rate and its current application in research is sustained as a new norm [1, 2]. Driven largely by the necessity to maintain operations and reduce in-person contact during the COVID-19 pandemic [3], the adoption of digital methods was often reactive and implemented with little prospective research to inform best practices [4].

**Table 1:** Digital methods commonly used in digitally enabled research

Digital method	Description	Application to potential and enrolled participants
Multi-media presentation	Presentation of research information using slides, videos, and pictures	Communicate study protocol information
Mobile application	Digital use of small, wireless devices such as a tablet or smartphone	App-based data collection, communication with the study team
Social media	Online networking platforms such as Facebook, X (formerly Twitter), and Instagram	Advertisement and recruitment
Electronic consent	Microsoft Word, Adobe PDF, and purpose-built software common (e.g. RedCAP, Medidata)	Electronic or biometric signature in place of a wet ink written signature
Web-based programmes	Web browser or smartphone applications	Data collection, participant interaction with research staff, study visit management
Virtual messaging	Digital messaging services including text messages, emails, and purpose-built applications	Asynchronous 1- or 2-way communication between research staff and participants
Wearables	Technology enabling real-time remote data collection (e.g. activity, heart rate, ECG)	Real-time or batch collection and monitoring of biometric data

In line with digital global and national health initiatives [5–8], the Department of Health and Social Care (DHSC) 2022–2025 strategy for research delivery aims to make digitally enabled research the new norm [9] leading healthcare and research organisations such as the National Health Service [10] and UK Clinical Research Collaboration (UKCRC) Clinical Trials Units (CTUs) to adopt technologies that support this digital transformation [11]. However, there is concern that these methods may hinder engagement in studies, particularly amongst underrepresented populations who are reported to use digital technology less, such as older adults, ethnic minorities, and those experiencing socioeconomic disadvantage [12–17]. The distinction between digital health interventions and digital research methods is a critical consideration in healthcare research. Digital health interventions are purposefully designed to improve health outcomes, potentially offering participants direct benefits and subsequent motivation for engagement (e.g. structured online rehabilitation programme). In contrast, digital research methods primarily serve as methodological tools for carrying out research protocols and data collection, where participants may experience minimal personal benefits, potentially resulting in reduced motivation for engagement (e.g. completion of a 6-month follow-up assessment).

Limited research has examined the intersecting factors that shape the use of digital methods across a study’s lifecycle. It remains unclear who might benefit or be disadvantaged from these methods and what factors might enhance or hinder engagement. Investigating the key drivers of engagement with digital methods across different population groups could inform strategies to improve representation in health research. This, in turn, could enhance the generalisability of findings and help address existing health inequalities. Whilst efforts are made to identify factors that influence engagement with different digital health interventions, leading to evidence-informed strategies to optimise their implementation [18, 19], digital methods used to conduct research related activities before, during, and after intervention testing (e.g. informed consent, data collection, research communications) remain unexplored.

Quantitative evaluations of digital methods’ impact on research participation have been inconclusive due to small sample sizes, varied study contexts, and inconsistent terminology and reporting [20–23]. Some report a high risk of selection bias (e.g. assessing attitude towards digital research in participants on a digitally enabled study) whereas others fail to report sample demographics [24], making in-depth inferences on population based impact of digital methods unattainable. Studies have reported differences in the demographic uptake of digital vs non-digital methods [25, 26]. The ADAPTABLE study [27] (*N* = 15,076), which is an interventional low vs high dose aspirin study, provided options for participants to use ‘internet’ or ‘non-internet’ methods to engage in the study and reported significant differences in the demographic characteristics between groups. The ‘non-internet’ group were on average 2 years older, had a higher proportion of females (38.9% vs 30.2%, *P* < 0.001), a higher proportion of Black (11.1% vs 6%, *P* < 0.001) and Hispanic (11.1% vs 2%, *P* < 0.001) populations, and more comorbidities (myocardial infarction, congestive heart failure, diabetes, *P* < 0.001) than the ‘internet group’. Whilst similar evaluations also report differences in demographic engagement with digital methods, the underlying mechanisms driving these observations remain poorly understood. Moreover, current evaluations approach their analysis with simplistic and singular population categories, which have limited applicability to real-world decision-making where any given participant has multifaceted and interacting identities.

Deeper insights have been attempted using qualitative research, but many studies have encountered limitations with surveys distributed online and biased samples from small, homogenous interview groups [11, 28, 29]. Some studies focused on a single digital method (e.g. informed consent) on a specific platform [30, 31], missing potentially important influencers to digital engagement through a person’s study journey.

Intersectionality, used in this study to acknowledge how race, class, gender, sexuality, ethnicity, nationality, and age interact to shape social phenomena, has received increasing attention with the rise of equality, diversity, and inclusion initiatives in health research [32]. Failure to consider the intersecting components that make a person’s identity, in addition to their research journey—especially when they are also receiving healthcare—creates challenges for research communities aiming to optimise a participant’s study engagement. This gap in holistic understanding hinders the ability to make informed decisions about whether, when, and how to adopt these technologies in practice, as well as understanding the potential consequences for different segments within a target population.

This study aims to understand the factors influencing engagement with digital methods used to conduct health research by exploring the experiences and perspectives of different intersectional groups of people (e.g. by age, gender, ethnicity). This study’s purpose is to contribute evidence-based insights for research communities who wish to advance research conduct practices.

## Methods

### Design

Qualitative semi-structured interviews were used to gain an in-depth understanding of the factors that influence engagement with digital methods in health research. The philosophical stance chosen for this study was critical realism [33]. This study was reported in line with APA Qualitative Design Reporting Standards (JARS-QUAL) and Consolidated Criteria for Reporting Qualitative Research (COREQ) reporting guidelines (Multimedia appendix 2 and 3, respectively).

### Study setting and recruitment

To ensure the data were grounded in lived experience of research participation and to be confident information power [34] would be sufficient across different demographic groups, up to 50 people who had taken part in UK health research were the target population. Patients were eligible if they were over 18 and had participated in UK interventional or non-interventional health research in the past 12 months and had provided consent to take part in the interview. Initially, participants were eligible if they were actively participating in research that offered at least one participant facing digital method; halfway through the study this was removed and participation experience extended to within 12 months to assist with diversifying the demographics of the sample. People were excluded if they had any self-reported mental or physical developmental impairment that impacted the use of digital methods; this population were anticipated to have unique experiences, which would be better served through separate exploration. Translational services were available for people who were not proficient in English; however, no one with experience of participating in health research who consented to take part in this interview study required linguistic support.

This qualitative study was signposted by research staff via Clinical Trials Units and Research Delivery Networks in community, primary, and secondary care settings and advertised to potential participants by digital (e.g. email) and non-digital means (e.g. flyers).

**Fig. 1:**
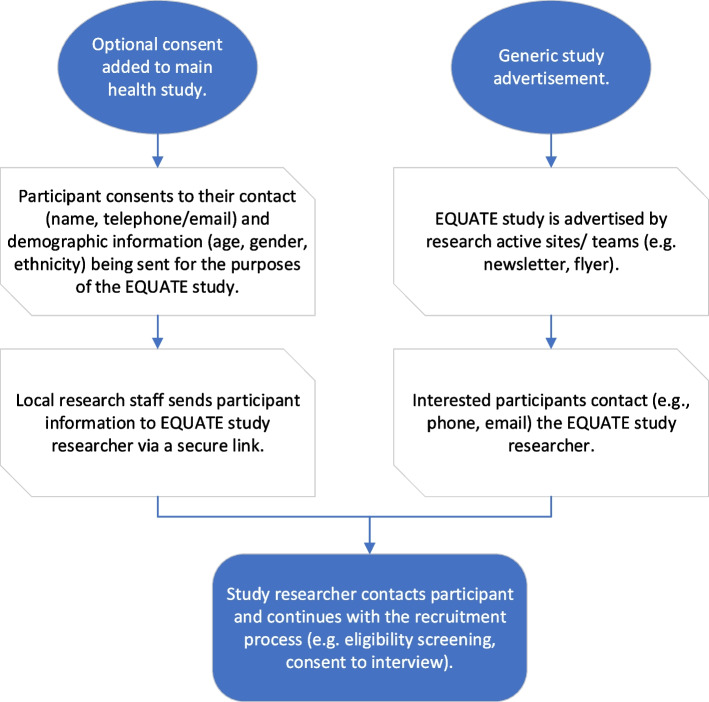
Study recruitment schema

Participants expressed an interest in being interviewed by optionally consenting to be contacted in the recruiting study consent form or by contacting CB directly by phone, email, or QR code (Fig. 1).

Those perceived to be most at risk of being inadvertently excluded from digitally enabled studies such as minoritised ethnicities, older adults, and people experiencing socioeconomic disadvantage were purposively oversampled through demographic information provided by study teams or self-reported information upon screening. Where possible, a participant’s chosen preference of contact in health research (e.g. email or post) was also used to inform the sample. All expressions of interest were responded to by phone or email by CB to screen for eligibility and answer any initial queries about the study. All participants received a copy of the consent form and information sheet by post or email before the interview and written or verbal consent was obtained. On completion of the interview, thanks were given, a £10 voucher of the participant’s choosing was provided, and a summary of findings was offered.

Interviews were paused periodically to review insights, sample size, and reflect on the diversity of recruited participants with specific focus on information power from ethnic minorities, older people, and people experiencing socioeconomic disadvantage.

In total, 50 interviews were completed with participants in Hampshire, Herefordshire, West Yorkshire, West Midlands, Cheshire, Wales, and London. Regional comparisons were not undertaken because of the variability in study contexts both within and between regions, the dispersed nature of our relatively small sample across primarily urban and suburban settings, and the study’s emphasis on individual participant perspectives rather than geographical trends. Two invitations were declined at screening due to lack of interest (Table 2). Interviews captured experiences in observational, qualitative, and interventional health research studies (Table 3).

**Table 2:** Study population

Characteristic	Participants, *n* (% of total)
Gender	
Female	22 (44%)
Male	28 (66%)
Age (years), median (IQR); min–max	62.9 (29) 19–87
Ethnicity	
White	35 (70%)
Black African	4 (8%)
White and Asian	5 (10%)
Bangladeshi	2 (4%)
Chinese	4 (8%)
Highest level of education	
No formal education	8 (16%)
GCSE (or equivalent)	12 (24%)
A levels (or equivalent)	10 (20%)
Undergraduate bachelor’s	10 (20%)
Higher than undergraduate	10 (20%)
IMD* score	
1–3	14 (28%)
4–6	17 (34%)
7–10	19 (38%)

*IMD* Index of Multiple Deprivation, based on residential postcode. 1 (most deprived) to 10 (least deprived)

**Table 3:** Study type participants took part in

Research design	Participants, *n* (% of total)
Qualitative	9 (18%)
Questionnaire study	7 (14%)
Observational	9 (18%)
Interventional	25 (50%)
Phase II—medicinal	4 (8%)
Phase II—medical device	6 (12%)
Phase III—medicinal	15 (30%)

### Data collection

Semi-structured interviews aiming to explore participant experiences and perspectives of digital methods began on 11 Oct 2023 and finished on 30 August 2024. Interviews followed a topic guide (Multimedia appendix 1) developed by CB and KB and were conducted by phone or video call (participant’s choice). Both video and telephone interviews were recorded using Microsoft Teams. The interviews lasted between 50 and 60 min. A female interviewer (CB; Doctoral NIHR Fellow and Qualitative Researcher with 10 years’ experience in conventional and digital patient-facing research delivery and research management) conducted all interviews. Overall, CB felt she held a balanced view on the research topic. Her experience working in a Clinical Trials Unit developed her empathy for the resource constraints faced by staff conducting research, driving many to seek efficiency. Additionally, her interviews and face-to-face delivery experience provided her with an understanding of the emotional and physical needs of research participants. Approximately two-thirds of participants with an ethnic minority background were familiar with CB through a previously completed community-based study; all other participants were not known before being interviewed but engaged in generic conversation pre-interview to build rapport.

### Data analysis

Recorded interviews were independently transcribed verbatim, pseudonymised by title using participant IDs, fully anonymised in text through the removal of names, areas, and organisations, and error-checked by listening back through the original audio file by CB, which also served to re-familiarise the data before coding. Reflective thematic analysis [35] was used to emphasise the researcher’s reflexivity, which given the previous and ongoing work within a clinical trials unit was seen as crucial to understanding the potential influence this had on the interpretation of the data.

This analysis approach also suited the context-rich, iterative, and interpretive flexibility required to address the research question.

Transcripts were not shared with participants. CB managed the data and led the coding and analysis of all interviews using Nvivo software (v14). A trial manager (NE) with master’s level experience in qualitative methods independently coded and analysed a subset of the interviews halfway through data collection. The goal of independent coding was to challenge assumptions, deepen reflexive insights, and highlight the most insightful interpretations. To further challenge emerging patterns, discussions were held with coauthors and some uncoded and anonymised excerpts of transcripts in line with developing themes were shared with patient and public representatives.

Except in two cases where data collection was paused to catch up and reflect on the progress of the study, data analysis occurred concurrently with data collection to inform iterations of the topic guide. Initial coding of the first five interviews noted points of interest and was refined through merging of similar codes which continued to evolve as more interviews were analysed. To assist with the interpretation of data and generation of themes from the large dataset, initial codes were recorded in a coding framework. The research remained open to new codes and concepts, especially where participants had characteristics not yet well represented (e.g. new age and ethnicity combinations).

Codes relevant to the research question and the interpretation of the codes content amongst different participants generated themes. The relationship between codes, themes, and the intersecting groups they appeared to be linked to was reviewed and deviant cases were sought to challenge interpretations [36].

## Findings

Three interconnected themes demonstrate how multiple aspects of a person’s identity and social position can create pathways of engagement towards or away from digital methods.

### Digital Positionality: The Interplay of Social Position, Personal Experience, and Identity

Each participant appeared to have several interacting factors that shaped their relationship with digital methods.

The interactions between migration status, language proficiency, and age appeared to create roles that led segments of this population to be a digital supporter or digital dependent.

Younger second-generation adults with bilingual language skills tended to feel high levels of confidence engaging with a variety of digital technologies, and on reflection attributed this to their experiences in the educational system and contemporary social interactions. People with these characteristics shared how they would commonly fulfil the role of language and digital mediator for older immigrants with limited English proficiency in a health research context.

*I know Punjabi. I explained the questionnaire, like I convert them. I say I explained it in my language so that they can understand.* (34, male, Pakistani).

Many older adults saw technology as something for younger generations; second-generation adults, especially those living with or near their first-generation parents, seemed to feel a greater responsibility to support less digitally literate elders. This increased sense of responsibility may have been due to the need to navigate additional linguistic and cultural challenges or the expectation within their culture to respect and support elders.

*I sent a video showing how turn your hot spot on..she calls me like 2 h later…screaming at me saying it’s not working. Where are you? Come home. And then obviously I’ll go a bit angry because I’m just trying to enjoy time with friends. I told her how to do it… it’s very annoying*. (22, male, White and Asian).

Socioeconomic status and accessibility needs were strong interacting influencers towards people’s experience with several different digital methods. Those with greater financial resources spoke of having navigated physical needs, such as poor vision or dexterity, by acquiring multiple devices such as smartphones and tablets with larger touch screens. The ability to pick a device perceived to best suit a task appeared to help establish positive feedback loops of engagement.

*Well, I haven’t actually picked up the phone because I’ve got Apple computer and Apple tablet and Apple phone, they all ping off at the same time when something comes in…. I prefer to have it on a bigger screen…* (58, female, White).

Conversely, participants experiencing economic constraints often relied solely on smartphones, with a couple also mentioning a lack of software access (e.g. Microsoft Office) and compatibility issues. The most common frustration shared was the need to zoom in and consequently scroll sideways along sentences and down through large volumes of text.

*I had to keep scrolling back to look at the questions and fill it in* (35, female, White and Asian).

Home laptop access was largely limited to participants with employer-provided devices who in this sample were less likely to be experiencing socioeconomic disadvantage or have unmet accessibility challenges.

Findings also revealed how a person’s household role could interact with access to social support and age to create a particularly strong combination of factors that influenced behaviour towards or away from digital methods adoption.

Many participants reported how they or another member of the household were primarily responsible for the digital tasks in the household. In older adults, digital tasks were typically taken on by men, leading older woman to appear at a particularly high risk of having digital anxiety and low self-efficacy which seemed to further perpetuate their dissociation with technology and the use of digital methods.

People who experienced a disruption to these household roles (e.g. loss or divorce) seemed to face compounding risk of non-engagement due to low digital skills, lack of sustained in-person support from friends or family, and a disinterest in adopting behaviours that conflicted with their identity.

*It [PC tablet] belonged to my husband…he was the computer one.* (72, female, White).

This identity driven dissociation with digital seemed to result in little motivation to pursue or retain training or support.

*Somebody showed me how to get on it once [NHS app] but I don’t use it. I don’t really want to know to be honest with you.* (77, female, White).

Professional role transitions between digital and non-digital job demands, and retirement, emerged as a cross-cutting factor that had a strong influence on enhancing or diminishing engagement with digital methods. Some retired participants who expressed a level of confidence engaging with digital methods seemed to maintain skills and habits formed in their workplace, whereas others, especially where there was no perceived social or household need, experienced a disconnection from their former digital self.

*I get up early and I get at it first thing in the morning, and I check my emails just like me when I was in my working days.* (78, male, White).

These findings reveal how initial engagement with digital methods can be influenced by a relationship formed by a complex interplay of practical resources, social roles, and personal identities.

### Power Redistribution in Research Relationships: Navigating Vulnerability and Agency

This theme explores how intersecting aspects of identity influence power dynamics across several digital methods, revealing how participants might navigate different communication methods in health research.

A combination of limited English proficiency and an unbalanced research staff-participant power dynamic was seen to create compounding motivators to engage with digital methods that enabled self-directed time to understand and process study information. It appeared that the largest challenges for this group were faced during in-person health research discussions, where participants seemed linguistically or culturally inhibited from seeking research related clarifications.

*.. as a foreign national, probably my understanding will be slightly less than the native speakers, but you wouldn’t like to look like you didn’t understand…* (37, female, other Asian).

A couple of participants with limited English language proficiency reported that verbal and written recruitment experiences were received in a way that did not help them feel able to easily understand the information being provided. Pre-conversation recruitment materials, particularly video-based resources with images, were a preferred first point of introduction to a study. Acquiring an initial understanding of the study was felt to preserve their sense of dignity during subsequent discussion with research staff. Face-to-face interactions with staff remained crucial for building trust; however, participants indicated a need for time to process the information post-conversation. The provision of digital written materials following verbal discussions enabled participants to easily store and customise the format of documents to facilitate better understanding (e.g. translation, creating space between text).

*I do have some sort of issue with reading.. what’s been very helpful for me when it comes to the complicated text, is that I break them down into paragraphs and then make that on a big screen, and then read. That helped me process the information better…there are so many accessibility technologies are available on Google.* (37, female, other Asian).

These findings highlight how digital methods can operate in a supporting capacity during the informed consent process; however, other participant accounts highlight how when used in other contexts digital methods can be perceived to protect or threaten a participant’s sense of control.

The intersection of socioeconomic position, gender, and professional identity shaped how participants negotiated their presence when using digital methods at home. For those without profession-derived digital identities, information exchange via video calls appeared to surface defensive behaviours due to insecurities relating to domestic space and self-presentation, with the latter being particularly present in women. These participants seemed to experience their home environments and at-home appearance as potential sites of judgement, revealing how video calls can unintentionally expose social hierarchies that would otherwise be protected during site-based visits or non-visual communications.

*You do feel a sense of power imbalance a lot of the time. Whereas over the phone, it’s just the voice on the other end… It felt like I was talking to another person and not someone with a station or a position.* (37, female, White).

Those with work-provided equipment and professional spaces at home often demonstrated a greater openness to video calls, whilst those relying solely on their mobile device which, in addition to the above-mentioned concerns, also caused ergonomic challenges (e.g. uncomfortable posture, balancing phone) reported favouring conventional phone calls for verbal communication.

There were a couple of cases where it appeared that a participant’s distance to the research team influenced information sharing behaviour. In one case, an older female participant described dreading research (questionnaire) phone calls. The participant appeared to have no rapport with the researcher and, due to what seemed to be provoked by a perceived social hierarchy, felt pressured to say what she thought the researcher wanted to hear. As a result, the imbalance in power dynamics appeared to provoke the provision of false information.

*I took two [pain relief tablets], but I told her [research staff] I took one simply because… it was like ‘you will do this won’t you?* (70, female, White).

In this case, the participant went on to share how she nearly left the study, and only stayed after being offered the option to provide data via postal questionnaires. For convenience, the participant would have preferred completing the questionnaires digitally, but (despite this being an option to others on the study) this was not offered to her. In this case, distance was seen to mitigate the potential influence of social desirability of participant-reported data.

Conversely, another participant seemed to use their physical and emotional distance from the research team to conceal a drug-induced rash and maintain her position on a trial. She suggested this would have been less likely to occur had she developed rapport with a staff member.

These findings indicate how digital methods have the potential to disrupt or exacerbate traditional social hierarchies depending on the context in which they are being used. They also highlight the complex balance that must navigate between ensuring proper safety oversight (traditionally achieved through in-person research visits) and the convenience that digital approaches may offer. Data accuracy presents a particular challenge—digital methods may reduce accuracy when participants lack a connection to the study or personnel running it yet simultaneously may enhance accuracy by reducing social desirability bias in participant responses. This multifaceted trade-off is likely to vary across study types, for example different considerations for high-risk interventional versus low-risk observational research.

### Trust assemblages: how intersecting identities shape multi-modal verification practices in research engagement

DeLanda’s assemblage theory [37] focuses on the concept of assemblages as collections of heterogeneous elements that come together to form functional wholes. These elements can be both material (e.g. physical objects) and expressive (e.g. social practices, language).

This theory is relevant to this theme as it helps to conceptualise how different intersecting populations carry out different combinations of digital, physical, and social actions, referred to here as assemblages, to verify trust in a research organisation, study, or team.

Regarding remote invitations, posted letters were commonly seen by all as a more personal mode of communication and, seemingly due to their greater resource cost and lesser use in society, tended to leave an impression of importance. For many, the combination of a trusted sender and materiality of posted letters functioned as an importance and trust signifier that transcended digital immediacy, suggesting how physical methods can retain symbolic power as part of trust formation. Despite this, younger adults tended to feel that due to a lack of spontaneous availability or convenience, physical letters, although gaining attention upon receipt, were less likely way to evoke a response compared to their digital counterparts.

Whilst institutional markers such as NHS logos or university affiliations formed a baseline of initial trust for many, participants’ own positions appeared to shape how they navigated deeper connections with the research. Participants, particularly those invited to longer term or interventional research, sought out personalisation through individual address and sign off. Older females and ethnic minorities especially seemed to desire information that would help them construct the identity of the researcher.

*A little letter of introduction… my name is XYZ. I’ve been studying at the university… something personal* (70, female, White).

The intersection of age and cultural identity appeared to produce unique trust dynamics. Older first-generation participants engaged in trust-building processes that commonly prioritised community structures, whereas their younger second-generation counterparts occupied a unique position of cultural mediation—possessing high digital confidence which gained them access to worldwide information whilst also relying on traditional community trust structures. These participants’ trust verification processes reflected this duality, combining independent digital verification with community-based validation, with the latter weighing more heavily if there had been a negative online encounter (e.g. social media scam).

*She [community leader] told me directly, would you like to come [to a study focus group]? I was like, yeah. Like, that meant a lot…* (22, male, White and Asian).

For these populations especially, these accounts of digital-cultural mediation reveal how multi-level trust building in research is increasingly a collective rather than individual process.

For most participants, acquiring a ‘visual anchor’ of the person or team that represented the research felt important for initial and sustained engagement in a study. Some patterns of preference between how this visual anchor was created appeared complex and depended on digital ability, social position, cultural expectation, personal traits, and study factors such as level of commitment perceived by a given study to the individual.

Although participants developed impressions and connections with the organisation, study, and researchers through various means, it was the in-person, face-to-face interactions that appeared to prove most decisive influencer to participant engagement. These physical encounters ultimately either confirmed or contradicted the impressions and feelings participants had developed either before or after these meetings.

*..whether it’s the person who offers to make you a cup of tea, whether it’s the young clinician who’s apologising for prodding you in places that normally wouldn’t have, you know it’s the whole atmosphere…and that seems to be enhanced obviously by resources.. the flow of information is really clear. Everything that I’ve given to them was taken with respect and the way that I’ve been treated has built up my trust in that process.* (69, male, White).

In a few cases where a relationship did not seem to have been established with research staff at an individual level, participants that appeared motivated by an altruistic and/or personal value to their contribution did continue participating in the study.

These trust assemblages reveal how conventional concepts of trust in digital research is not a linear process but as a taxonomy of verification practices influenced by participants’ intersecting positions which are enacted through multiple channels.

## Discussion

### Key findings

This qualitative study uncovers how intersecting identity, cultural, and social factors can shape engagement with digital research methods, findings which align with an intersectional approach to understanding participant experiences.

Intersectionality was first introduced by Kimberle Crenshaw in the context of law and social justice to explain the oppression of African-American women [38]. Intersectionality has since been increasingly recognised and applied across various disciplines including health [39, 40], sociology [41, 42], and digital technology [43, 44]. Applied as a framework, intersectionality highlights how multiple social categories, such as gender, socioeconomic status, and race, interact to form social experiences that are distinct from the sum of their parts.

**Fig. 2:**
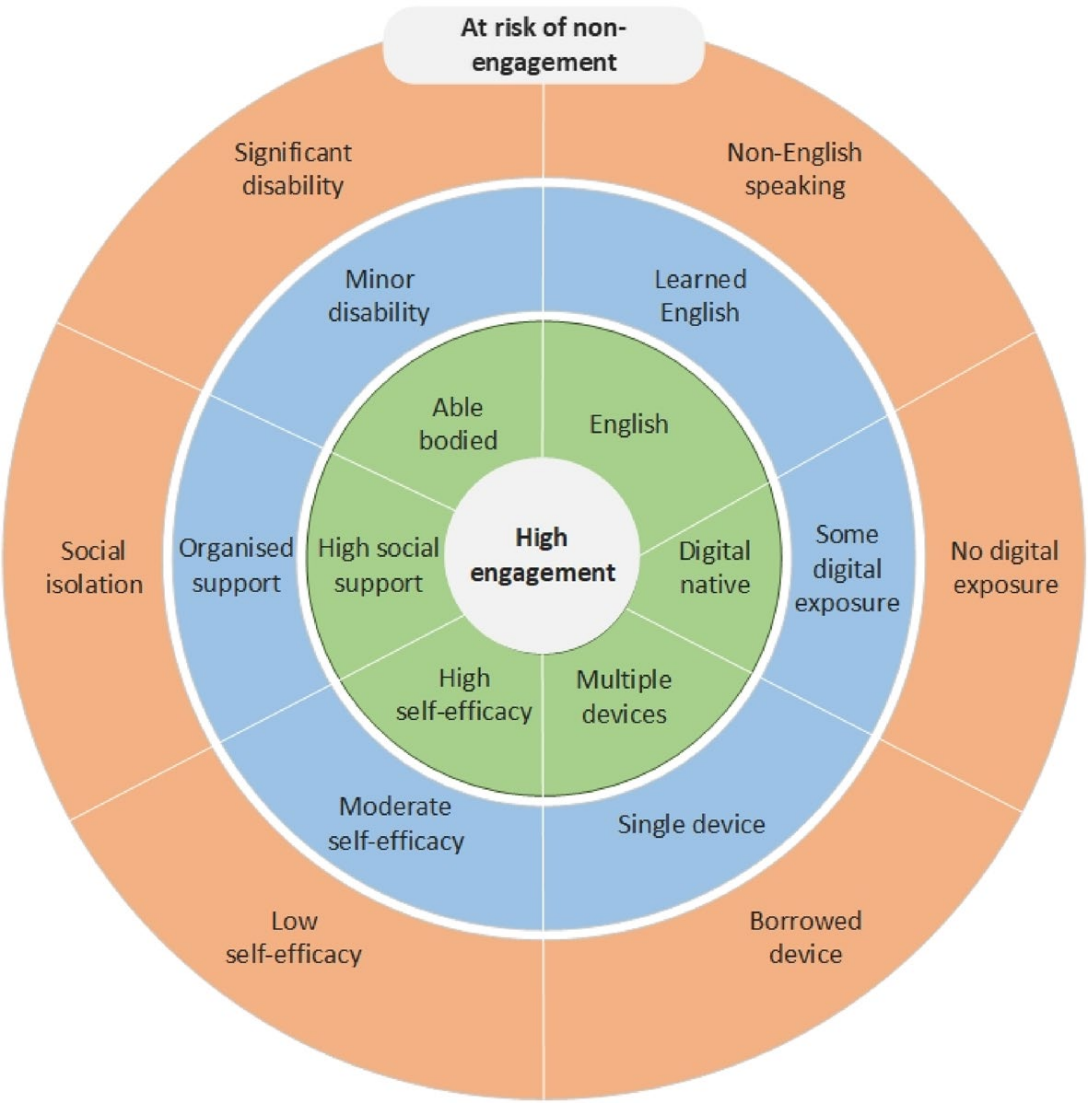
Wheel of digital methods engagement in health research (adapted from Wheel of Power/Privilege [45])

In the context of digital methods, this study has revealed how initial and ongoing engagement are shaped by multi-layered and complex interdependent factors that are not based on isolated and superficial characteristics (e.g. age, gender, ethnicity). Moreover, findings from this study indicate an overlap with people who are more or less likely to engage with digital methods and proposed power and privilege dynamics that shape who typically has resource access and who’s voices are heard in our society (Fig. 2).

This overlap suggests that a person’s social position influences their engagement with digital research methods. In combination with the critical impact of the third-order digital divide, the divide that considers the disparities between the benefits received by those who use digital technology and those who do not presents a critical challenge for health research. Implementing digital methods into health research without careful consideration and best practice guidance represents a pivotal moment that could lead to further exclusion of the most marginalised people—and is a reality we may only recognise in hindsight.

The concept of ‘digital positionality’ was introduced to help highlight how an individual’s personal relationship with technology is influenced through social position, life transitions, and personal identity. These intersecting factors extend beyond many traditional technology adoption or digital divide frameworks towards deeply embedded influencers towards or away from initial engagement with digital methods.

It also became apparent that digital methods can function to heighten feelings of control and power but also vulnerability in research communications. Whilst digital materials can support accessibility, agency, and manage power dynamics, video calls in particular can expose social hierarchies. This contrast appears to be especially apparent at the intersection of gender, socioeconomic status, and professional digital identity.

‘Trust assemblages’ shows how research participants construct trust through complex configurations of verification practices that span digital, physical, and social domains. Drawing on DeLanda’s assemblage theory and extending on existing work on trust formation for health research [46–48], this study shows how trust in health research is not developed as several singular strategies but through dynamic combinations of potentially accumulative and hierarchical processes that participants actively conduct based on their intersecting social positions, digital skills, and personal experiences. To advance existing practices, research communities should consider a more nuanced thinking of how global and study-specific trust markers might operate in concert, and how spreading these trust markers across digital, material, and social channels might be required to optimise diverse engagement with health research.

Findings from this research suggest that digital methods could inadvertently encourage or discourage research engagement and that compounding factors amplify pathways of advantage and disadvantage, a finding in line with several key theories such as intersectionality theory [49], digital capital theory [50], and fundamental cause theory [51].

The interacting factors that create these pathways extends on traditional concepts of digital technology as proposed in models such as technology acceptance model (TAM) and unified theory of acceptance and use of technology (UTAUT) series, which focuses on individual level anchors such as self-efficacy. Whilst previous research highlights how singular demographics such as age or ethnicity are reported to lead to higher or lower engagement with digital technology both in and outside of research, our findings reveal a more complex interplay of factors involving social and professional roles, life transitions, and personal experiences that shape the influence of engagement in research. This aligns with Husain et al. [30, 52] work on population disparities and e-consultations and adds the crucial study of how intersecting factors across diverse and research experienced populations can influence engagement with various digital research methods.

### Strengths and weaknesses

The study captured a diverse range of study types, disease areas, and participant perspectives, including those that are commonly underrepresented in health research. Overall, 30% of participants were from ethnically minoritised groups, 46% were experiencing higher levels of deprivation compared to the national average, and 42% were aged 65 or older. The intersecting group with the least participants were older adults from ethnic minority backgrounds, and people who were not proficient in the English language, potentially missing unique insights from these groups. It is acknowledged that the participants in this study hold a higher education level than that of the average educational attainment in England and Wales (50% vs 33.8% with A level education or higher) [53], and that the practices adopted in this study (e.g. remote interviews) could impact the willingness of some people to take part in this study.

The study approach addressed both initial participation and ongoing engagement, providing an in-depth and comprehensive view of factors influencing engagement with digital methods in different strata of the population.

Although interviewing people with research experience provided in-depth data, this focus could restrict the transferability of findings to people who have not taken part in research.

Most ethic minority participants interviewed in this study were known to CB through a community-based study. The implications of this are unknown, being known might have reduced perceived power dynamics and facilitated open conversation; however, there is a risk of social desirability.

This study included people with experience in health research from several broad demographic groups such as age, socioeconomic status, and ethnicity across a range of disease areas, suggesting a representative sample compared to other studies that have recruited homogenous populations with no in health research experience to draw on. Also, unlike some studies that focus on technology acceptance or uptake, this research was able to uncover the in-depth interplay of intersecting factors that influence engagement across diverse populations.

### Implications for research practice

**Fig. 3:**
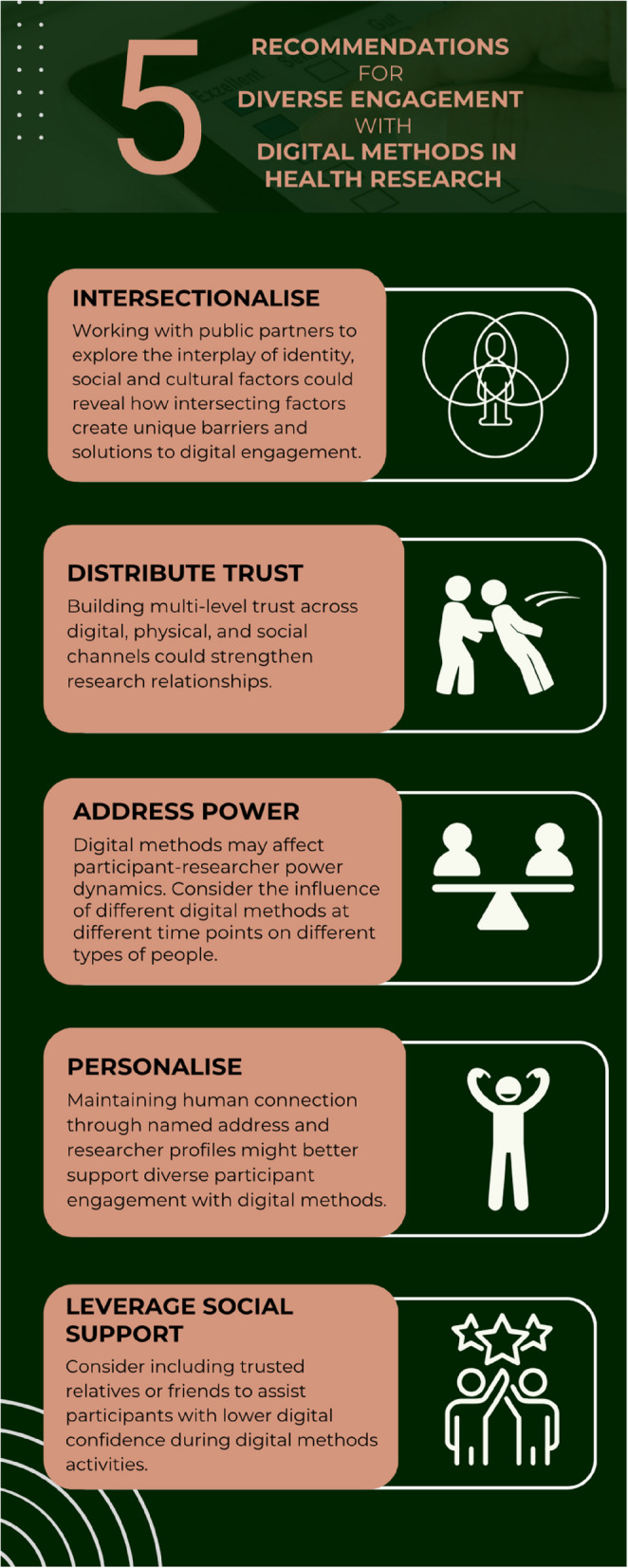
Recommendations for diverse engagement with digital methods in health research

The findings from this study inform a more advanced and intersectional way of thinking towards the design and implementation of digital methods in research. Research communities should move beyond the pursuit of assessing the impact of digital or non-digital dichotomies on singular and simplistic population demographics in pursuit of approaches that consider how different combinations of intersecting positions might influence engagement with digital methods and if and how they should be offered at different points of a participant’s research journey. Specifically, in addition to access and skills which can be overcome with device provision and support, researchers should consider how their chosen methods of interacting with underrepresented segments of a target population might align with or disrupt social roles and identities, and whether alternative methods or supportive strategies should be used (Fig. 3). Theories pertaining to multiple disadvantages should be applied into future literature to support the in-depth understanding and optimisation of research practices.

The findings on power dynamics reveal how digital materials can support understanding and dignity, but also how video calls might make some participants feel exposed in their living environment and ergonomically uncomfortable, especially for participants who do not have IT equipment and a dedicated space to professional working from home. In such cases where video calls are being considered, offering an alternative option could enhance participant agency and dignity in participant-researcher relationships.

### Future directions

Several questions remain unanswered, including the impact of trust assemblies and humanised systems influence engagement over time, particularly as new digital technologies emerge. There is also a need to understand how digital distance might influence data quality and participants’ ongoing levels of satisfaction.

In the interest of the larger picture, future works should also explore the influence of participant experiences to participation in future research.

Finally, there is scope for developing practical tools and frameworks to help researchers assess and respond to the intersectional dynamics identified in this study. This might include developing guidelines for intersectionality aware digital method selection and implementation.

## Conclusion

This study helps to advance our understanding of how intersecting factors can shape engagement with digital research methods. The first theme focused on ‘digital positionality’, uncovered how engagement with digital methods can be shaped by complex interactions between social position, life transitions, and identity performance—extending beyond traditional frameworks of digital access and skills.

Our work also highlighted how different digital methods can function as tools of empowerment and sites of vulnerability in research relationships, shifting the dichotomous focus from digital and non-digital comparison, to when and how it can be applied to help participants maintain agency and dignity. The concept of ‘trust assemblages’ provides a new understanding of how participants actively construct trust through multiple, concurrent channels shaped by their intersecting social positions.

These findings have significant implications for research practice. They suggest the need for more nuanced, intersectional approaches to digital method implementation that consider not just accessibility, but how methods align with or potentially challenge participants’ social roles and identities. Traditional approaches to digital research engagement that focus primarily on access, skills, and superficial and singular demographics may inadvertently reinforce existing patterns of advantage and disadvantage.

As research increasingly embraces digital methods, understanding these complex dynamics becomes crucial for facilitating participation across diverse populations. Future research should focus on developing practical frameworks for implementing these insights and exploring how different trust assemblages might influence long-term engagement and data quality.

## Supplementary Information

Additional file 1. Multimedia appendix 1 Topic guide

Additional file 2. Multimedia appendix 2 APA JARS-QUAL

Additional file 3. Multimedia appendix 3 COREQ guidelines

## Data Availability

The datasets generated and/or analysed during the current study are not publicly available to protect the identity of the participants but are available from the corresponding author on reasonable request. The standards for reporting qualitative research and topic guides used to inform the interviews are available as supplementary material. Please direct all data enquiries to the corresponding author.
