# Supplementary material for: The role of intersectionality in shaping participant engagement with health research through digital methods: findings from a qualitative study
Source: Trials. 2025 Jun 21;26:218. doi: 10.1186/s13063-025-08929-0 (PMC12182665; doi:10.1186/s13063-025-08929-0)
Supplement: Supplementary file 1 — Additional file 1. Multimedia appendix 1 Topic guide [file 13063_2025_8929_MOESM1_ESM.pdf]

Title: Barriers and facilitators to EQUitable pArticipation and engagement in research using digiTal mEthods: A qualitative exploration with the public, patients, and staff

Work package 1 Topic Guide

1. Can you tell me about the research that you're taking part in as a participant?

   Prompt: Establish disease area, how were they introduced, when they took part

   1a. How were you introduced?

   1b. Have you taken part in research before?

   1c. How would you prefer to be made aware of opportunities to take part in research?

2. What were you asked to do?

   2a. What ways did you share information as part of the research? E.g. phone call, paper, online

   2b. How would you describe your experience with X? What influenced your experience?

   2c. Did you need any extra information to engage with X?

   2d. Where were you when you completed the research activities? *In one go?*

   2e. Was this done with staff, friends, or family?

3. Were you given any alternative options to provide the information?

   3a. How would you have felt if you were presented with different options to provide information? / What options were provided?

   3b. What would your preference be? Why?

   3c. What's sorts of emotions did/ would you experience if you were asked to interact with a member of the research team over digital vs in person.

   3d. How would you most likely be interacting with friends and family week to week?

4. Can you tell me about any security or privacy concerns you might have had?

   4a. Was there anything that made you feel that way?

   4b. What could have made you feel differently?

   4c. Can you tell me about any times when you might have felt hesitant about engaging in online communication because of security concerns?

5. In general, day to day, how do you use digital technology?

   Note: get an idea of digital skills competency level

   5a. Can you recall when you were introduced to digital technology?

   5b. Can you tell me about any time you've used computers or in a job?

   Prompt: task being completed, confidence using, training received

   5c. Have you experienced any difficulty using digital technology in daily life?

   5d. What would you say has allowed you to keep up with technological changes over the years?

   5e. What did you do? / What would you do? *Preferred way of receiving support?*

   5f. Which family/ friends would you go to if you needed technological support?

6. How did you experience any ongoing communication in the research?

   6a. How did you feel about that?

7. How does a video call feel compared to meeting a researcher in person?

   7b. How different would you feel having seen a picture of the person on some information you'd received vs not?

   7c. If you were speaking to a research nurse because they wanted to ask some questions about your health, how would you most prefer to talk to them? Why?

8. What would your initial thoughts be if all research activities took place at research/hospital sites?

   8a. What would make you feel like your participation was valued in the scenario where you were travelling and seeing research staff in person?

9. What would your initial thoughts be if the research needed you to use only digital methods to take part away from sites e.g., from the home?

   9a. What would make you feel like your participation was valued if you had no in-person contact?

<u>Did not use digital methods</u>

1. How have [non-digital choice mentioned above] helped you to participate in research?
2. In what ways have [non-digital choice mentioned above] hindered your ability to participate in research?
3. Has anything stopped you from completing trial visits or activities?
4. Can you tell me about any situations or scenarios where you might find it helpful to use digital technology for research activities?
5. Can you tell me about areas or times you feel digital should not replace in-person visits?

Using digital methods

1. What was your first impression of the platform/website?
2. What was your overall experience of using x?
3. How has x impacted your ability to participate in research?
4. Can you tell me about any difficulties using x?
6. Was there anything you particularly liked?
7. What other areas would you like to see digital methods used in research?
8. Can you tell me about any situations or scenarios where you might prefer the use of pen and paper for trial activities, rather than digital methods? Why?
9. Can you tell me about any situations or where you might prefer the use of in-person visits for trial activities, rather than digital methods? Why?

10. If you were deciding about whether to take part in a study, what information about any technology use would you like to know upfront?

    Prompt: When and how would you like that information

11. Are there any other thoughts or experiences you'd like to share?
